# Supplementary material for: Rotations improve the diversity of rhizosphere soil bacterial communities, enzyme activities and tomato yield
Source: PLoS One. 2023 Jan 12;18(1):e0270944. doi: 10.1371/journal.pone.0270944 (PMC9836298; doi:10.1371/journal.pone.0270944)
Supplement: S1 File — (DOC) [file pone.0270944.s001.doc]

|  |
| --- |
| **Microbial diversity in 3 soils (16S)** |

**
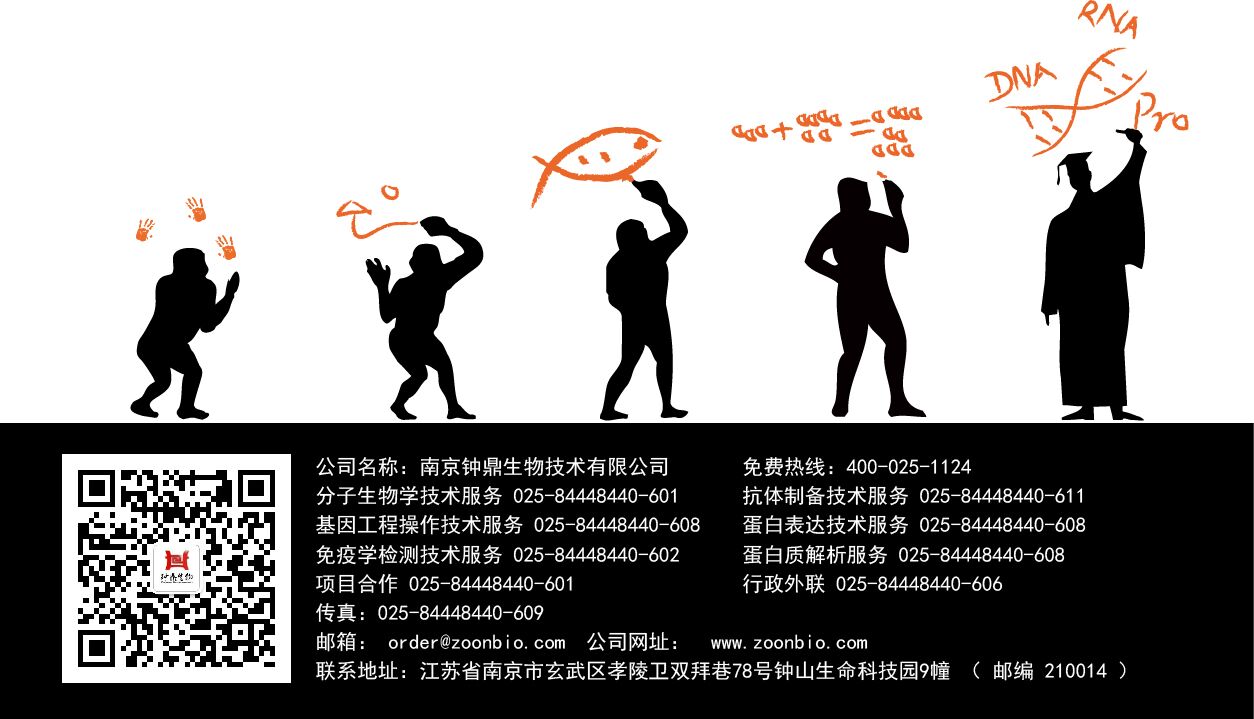

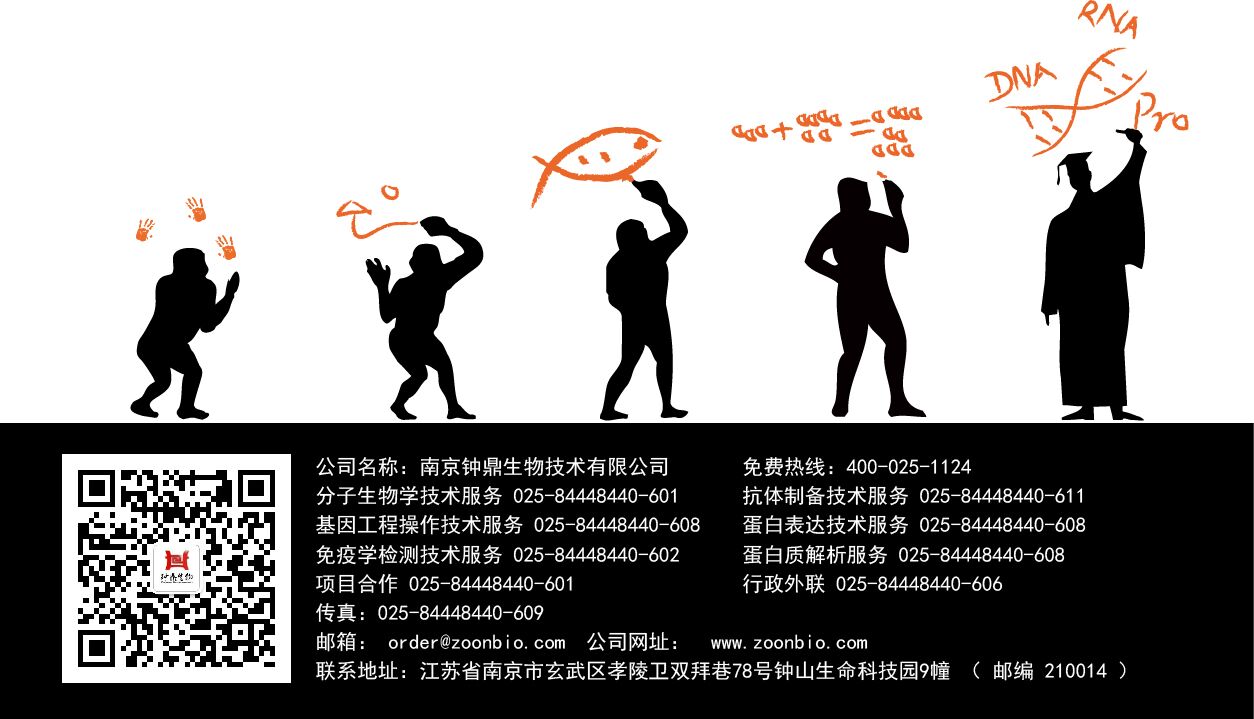
**

**
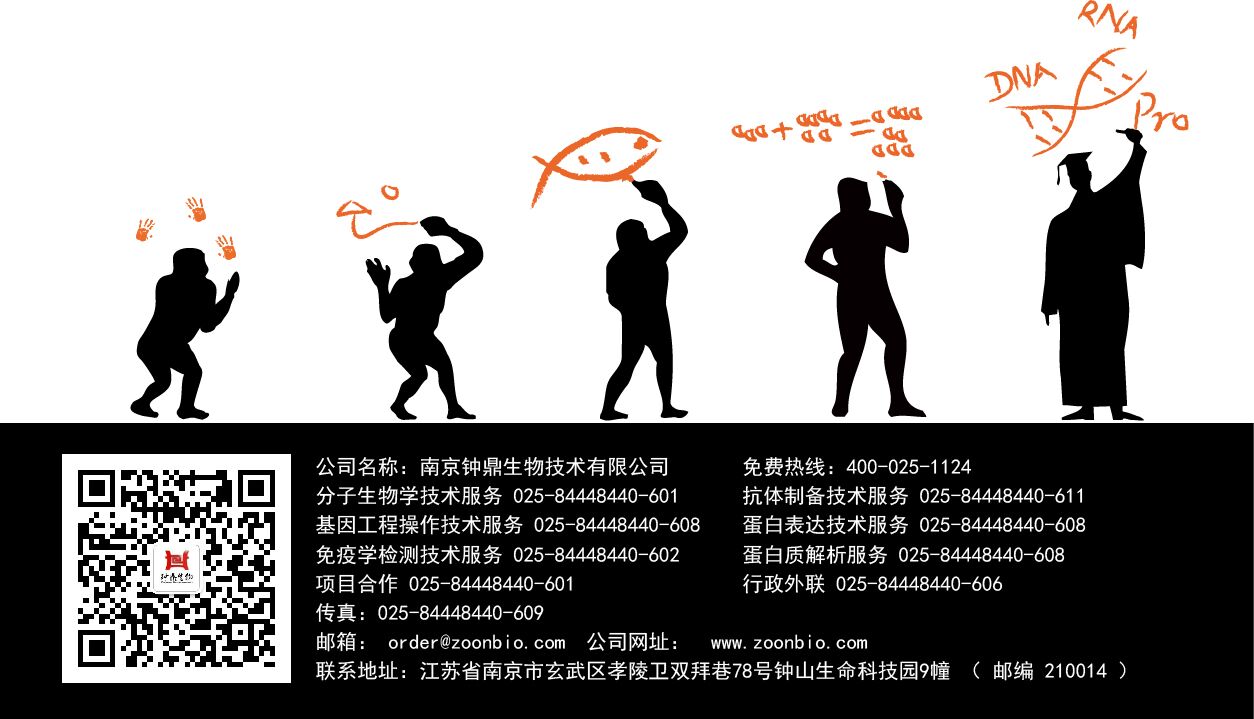

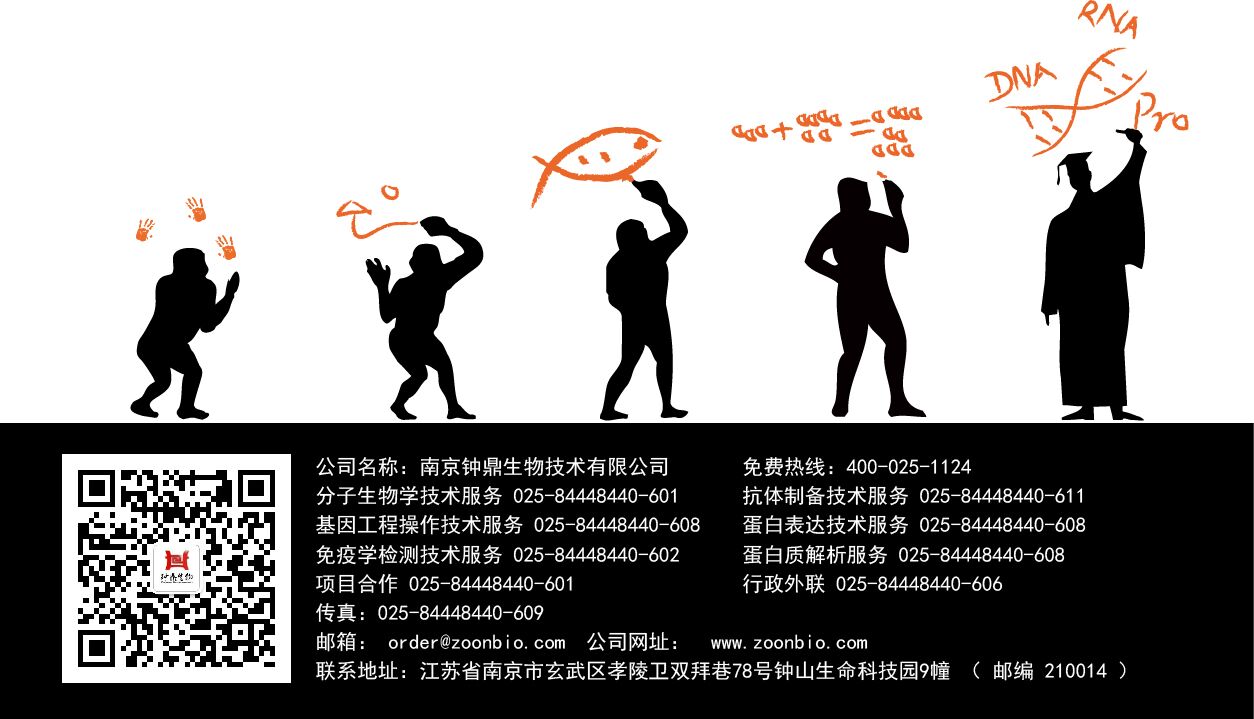
**

**
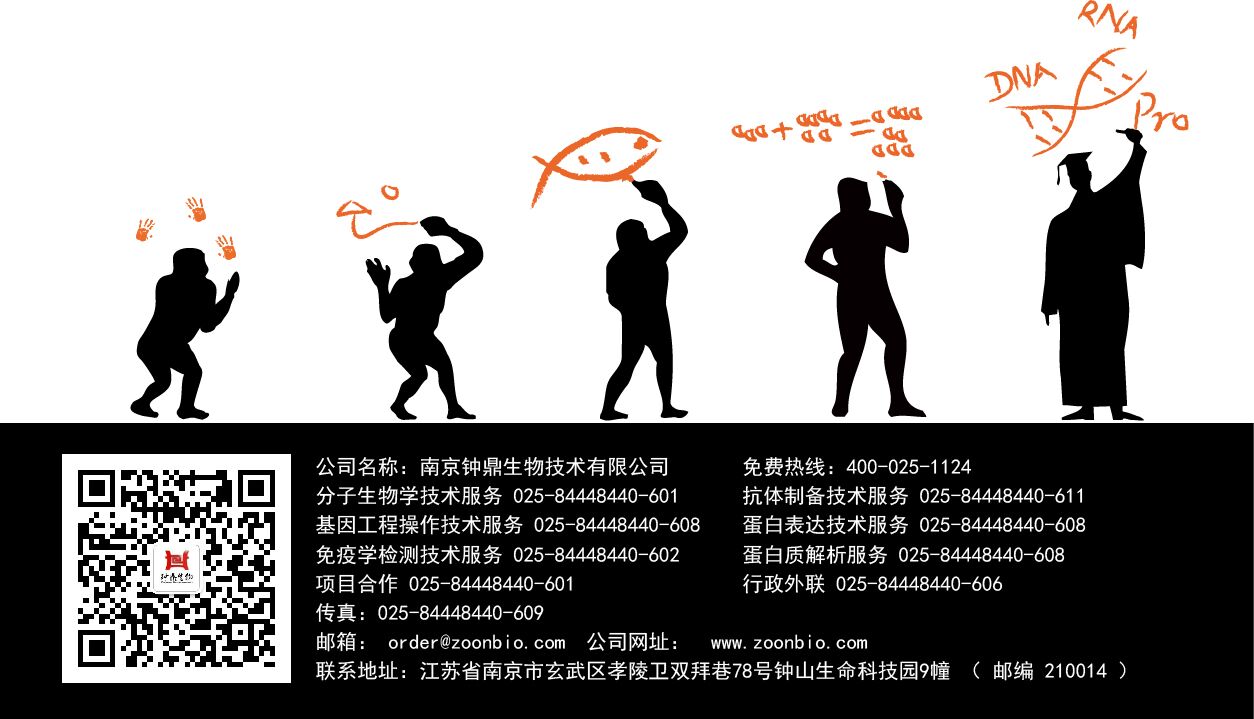
**

**Table S1. Sample data and OTU statistics.**

| **Sample name** | **Amplicon _type** | **Clean _tags** | **Effective _tags** | **Singleton** | **Singleton(%)** | **Chimeras** | **Chimeras (%)** | **otus** |
| --- | --- | --- | --- | --- | --- | --- | --- | --- |
| TY1 | 16S | 205957 | 168816 | 31746 | 15.4139 | 5395 | 2.6195 | 2673 |
| TY2 | 16S | 224549 | 194608 | 26082 | 11.6153 | 3859 | 1.7186 | 3112 |
| TY3 | 16S | 213453 | 174314 | 33330 | 15.6147 | 5809 | 2.7214 | 3271 |

**Table S2. OTU statistics of samples after drawing.**

| **Sample** | **OTU_Reads_Num** | **OTU_Num** |
| --- | --- | --- |
| TY1 | 168816 | 2673 |
| TY2 | 168816 | 3074 |
| TY3 | 168816 | 3266 |

**Table S3. Statistical results of sample Alpha diversity.**

| **alpha_name** | **chao1** | **observed_species** | **PD_whole_tree** | **shannon** | **simpson** | **goods_coverage** |
| --- | --- | --- | --- | --- | --- | --- |
| TY1 | 802.0111 | 772.0000 | 158.5981 | 5.0827 | 0.9145 | 0.9992 |
| TY2 | 995.8906 | 979.0000 | 192.8330 | 6.6566 | 0.9670 | 0.9995 |
| TY3 | 1064.8261 | 1047.0000 | 221.9722 | 7.1409 | 0.9784 | 0.9996 |

**Table S4. Alpha diversity index difference test.**

| **alpha_name** | **chao1** | **observed_species** | **PD_whole_tree** | **shannon** | **simpson** | **goods_coverage** |
| --- | --- | --- | --- | --- | --- | --- |
| p_value | 0.3679 | 0.3679 | 0.3679 | 0.3679 | 0.3679 | 0.3679 |
| Mean (TY2) | 995.8906 | 979.0000 | 192.8330 | 6.6566 | 0.9670 | 0.9995 |
| Mean (TY3) | 1064.8261 | 1047.0000 | 221.9722 | 7.1409 | 0.9784 | 0.9996 |
| Mean (TY1) | 802.0111 | 772.0000 | 158.5981 | 5.0827 | 0.9145 | 0.9992 |
