# Supplementary material for: Rotations improve the diversity of rhizosphere soil bacterial communities, enzyme activities and tomato yield
Source: PLoS One. 2023 Jan 12;18(1):e0270944. doi: 10.1371/journal.pone.0270944 (PMC9836298; doi:10.1371/journal.pone.0270944)
Supplement: Attachment — Submitted filename: Response to Reviewers.docx [file pone.0270944.s003.docx]

Response letter

Dear Editors and Reviewers,

Thank you for your letter and the reviewers’ comments concerning our manuscript entitled “Rotations improve the diversity of rhizosphere soil bacterial communities, enzyme activities and tomato yield” (ID: PONE-D-22-17572). These comments are all valuable and greatly helpful to revise and improve our paper. Based on the comments, we carefully made modifications. The changes are highlighted in red in the text. We gave responses to the reviewers’ comments one by one as follows:

**Reviewer #1:**

**1. Comment: The paper is interesting but there are few points that should be revised before publication. The authors should** **indicate** **which statistic test they have used to analyze the results (indicate p value also). In general the paper is written in a good English, but more references could be added to support the text. In particular, references in English would add value to the paper. There are** **ten references in Chinese, please find and include other references in English** **to support your sentences.**

Response: Thanks for your suggestions. The statistical tests used to analyze the results were added and the corresponding *p* values were provided. (Lines 198-199, 279-283, 292-296, and 310-312)

Several references in Chinese were removed, and 13 English references were added. (Lines 521-540, 575-577, 589-591, 602-604, 618-621, 644-652)

2. **Comment: In the discussion section,** **you should not repeat the results without further discuss them. I think you** **should revise the discussion** **section trying not to repeat only the results (for example, have a look at lines 311-315)**

Response: Thanks for your comments. We rewrote the Discussion referred to other researchers ’studies. The additions are marked in red in the paper. (Lines 321-323, 325, 329-333, 339, 343-350, 359-361, 364-370.)

3. **Comment: Keywords: I suggest to use keywords that are not included in the article title. In this way it is probable that more people are reading your paper.**

Response: We revised the keywords as suggested. (Line 42)

**4. Comment: Line 31: “Lysobacterium” is rarely used in the literature. In order to avoid misunderstandings I suggest to replace it with the more used and known “Lysobacter”.**
Response: We replaced “Lysobacterium” with “Lysobacter”. (Line 31)

**5. Comment: Line 74: please add references about tomato-rice and tomato-xerophyte crop rotation.**

Response: We added 3 references based on the reviewer’s comments. (Lines 521-532)
**6. Comment: Lines 75-77: add references to support this sentence.**
Response: 2 references were added. (Lines 533-540)

**7. Comment:** **Lines 158-159** **Specify the concentration of the sodium phenol solution and of sodium hypochlorite solution used.**

Response: We specified the concentrations of the sodium phenol and sodium hypochlorite solutions. (Line 164)
**8. Comment: Figure 4: if possible, please** **insert a bigger legend to the figure. The current legend is too small and difficult to read.**

Response: A bigger legend has been added to Figure 4. (Line 261)

**9. Comment: Lines 258-259: “TY3 had the highest level of activity.” What are you referring to in this sentence? Are you referring to catalase or sucrase? Please specify.**

Response: We are sorry for this confusion. It meant that TY3 had the highest level of catalase activity. It was changed in the text (Line 270)
**10. Comment: Lines 260-261: “[…]** **i****ndicating that the arrowhead-tomato rotation could increase soil fertility.” I think that this sentence should be part of the discussion session.** **Moreover, you should include more data supporting this sentence to be able to arrive at this conclusion.**

Response: This sentence has been moved to the Discussion. This conclusion was obtained based on the an analysis of Figure 5. We rewrote that part in the Discussion. (Lines 371-373, 375-377)

**11. Comment: Lines 267-269: These sentences should be part of the discussion section.**

Response: We moved these sentences to Discussion. (Lines 359-361, 368-370)

12. **Comment: Table 2: Please specify what are the numbers in the table** **and if they have a unit of measurement. In addition, I suggest** **to represent the table graphically. This would help the reader to understand quickly the results. Please, specify also the statistic test you used to analyze the data.**

Response: We made this correction based on the reviewer’s comments. The numbers in the table and their units of measurement have been noted. In addition, the table was represented graphically along with the statistical test that was used to analyze the data. (Lines 279-285)

**13. Comment: Figure 5 and Figure 6: Please specify which statistic test you use to analyze the data.**

Response: We made this correction based on the reviewer’s comments. The statistic test was added in the Figure 5 and Figure 6. (Lines 279-283, 292-296)

**14. Comment: Figure 6: What is the difference between (e) and (f) ? They are both “Total soluble sugar content”.**

Response: We are sorry for this mistake. Figure 7(f) has been modified. (Line 306)

**15. Comment: Line 325: replace “benefit” with** **“beneficial”.**

Response: “benefit” was replaced with “beneficial”. (Line 351)

**16. Comment: Lines 341 - 345: Can you** **find references to support these sentences that are not in Chinese language?**

Response: 1 English references to support these sentences were added. (Lines 629-631)

**17. Comment: Lines 356 – 357: “Nitrate is an important quality indicator of vegetables.”** **Please include references to support this sentence.**

Response: 1 references were added to support this sentence. (Lines 644-646)

**18. Comment: Lines 361 -363:** **This sentence assumes that sucrase is an indicator of soil fertility. Please include references that support your assumption (possibly** **in English).**

Response: References 40 and 55 were added to provide evidence for the sentence“assumes that sucrase is an indicator of soil fertility.”Both references are in English. (Lines 578-581, 629-631)

**19. Comment: Lines 367 -368: I do not understand how this sentence correlates with the rest of the text. Please rephrase and add a reference in English.**

Response: The sentence was rephrased and the 60 reference in English was added (Lines 399-400, 647-649)

**We wish to profusely thank you for your helpful comments.**

**Reviewer #2:**

**1. Comment: ABSTRACT
Lines 24-26: …" tomato-tomato continuous cropping (TY1), eggplant-tomato (S. melongena) rotation (TY2) and arrowhead-tomato (Sagittaria trifolia-Solanum lycopersicum) rotation (TY3)." Please standardize the descriptions. For example: tomato-tomato (Solanum lycopersicum) continuous cropping (TY1), eggplant (Solanum melongena) -tomato rotation (TY2) and arrowhead (Sagittaria trifolia)-tomato rotation (TY3).**
Response: We made this correction based on the reviewer’s comments. (Lines 24-26)

**2. Comment: Lines 37-39: "Altogether, compared with the tomato monoculture, the rotations of tomato with eggplant and arrowhead shifted the rhizosphere bacterial communities and improved the yield and quality of the vegetables." Does the increase in yield refer to all three species.**

Response: Yes. Compared to the tomato monoculture, both the rotations of tomato with eggplant and arrowhead can improve the yield of the tomato. (Line 39)

**3. Comment: Experimental design
It would be very interesting to insert some pictures of the crops. Is it possible?**
Response: It is a good suggestion. Unfortunately, we did not save photographs from the study.

**4. Comment: Line 128: …" Among them, arrowhead was grown in moist culture." What do you mean by moist culture? In hydroponics? Also, it would be helpful to have pictures of the growths.**
Response: Moist culture does not indicate hydroponics. It means that the soil water content was maintained at 90% when the plants were cultivated, and no water is visible on the surface. Please find the picture below.

**5. Comment: Soil sampling**

**From the description of the sampling, it seems to me that the rhizospheric soil was considered but not the roots. Haven't you considered evaluating endophytic bacteria as well?**

Response: This is a good suggestion, but for this study, we just considered the rhizosphere soil. Our next study will examine the endophytic bacteria in depth.

**6. Comment: RESULTS
Line 244: "3.3. Distribution curves of soil microbial abundance among different planting modes". Perhaps the paragraph number should be removed?**

Response: The paragraph number was removed. (Line 255)

**7. Comment: Fig. 4: The legend at the top right in figure 4 is too small and hard to read.**
Response: The figure 4 was changed with a clear legend. (Line 261)

**8. Comment: Fig. 6: The graphs e and f (Total soluble sugar) appear to be a duplicate of the same data. Is that so?** And also, I suggest better detailing the caption.

Response: Figure 7(f) has been modified, and we provided more detail to the caption based on the reviewer’s comments. (Line 306)

**9. Comment: Final comment:
Even if English is good, I suggest a language revision to fix some problems.**

Response: We made these corrections based on the reviewer’s comments.

**We wish to profusely thank you for your helpful comments.**

**We tried our best to improve the manuscript and made changes in the manuscript. These changes will not influence the content and framework of the paper. In addition, we did not list the changes here and instead marked them in red in the revised paper.**

**We earnestly appreciate the thorough work of the editor and reviewers and hope that the corrections will meet with approval.**

**Once again, thank you very much for your comments and suggestions.**

**Best regards!**

**Yours sincerely,**

**Cui Feng, Xiaosan Jiang**

**E-mail:** [**20142202@jaas.ac.cn**](mailto:20142202@jaas.ac.cn)**, gis@njau.edu.cn (XJ)**
